# Pneumonia

**Published:** 1884-10

**Authors:** William Pepper

**Affiliations:** Provost and Professor of Theory and Practice of Medicine in the University of Pennsylvania


### BUFFALO

### Medical and Surgical Journal.

VOL. XXIV. OCTOBER, 1884. No. 3.

### Original Communications.

### PNEUMONIA.

A CLINICAL LECTURE DELIVERED AT THE HOSPITAL OF THE UNIVERSITY OF PENNSYLVANIA.

BY WILLIAM PEPPER, M. D., LL. D.,
Provost and Professor of Theory and Practice of Medicine in the University of Pennsylvania.

REPORTED BY WILLIAM H. MORRISON, M. D.

GENTLEMEN—The patient now before you is convalescing from an attack of pneumonia. I showed him to you one week ago on the fourteenth day of his attack, completely apyretic. He has not come up after this attack as quickly as we should like to have seen him. His past history has not been altogether satisfactory. In the first place, we find that he is the subject of constitutional syphilis, and, in addition, he has been exposing himself. When seized with pneumonia, he was not in a good state of health, and this has undoubtedly retarded convalescence; for he has been completely free from fever for ten days, with a pulse about normal and respirations not over twenty per minute; nor have the physical conditions progressed as rapidly as we desired; while the critical fall of temperature, the failure to rise, the slow pulse, the easy respiration, the tranquil face and the return of appetite, indicate that the process is practically at an end. There are still traces of infiltration along the anterior border of the right lung, showing that the elements of the tissues do not throw off all traces of the morbid action and return to the healthy state, but that the morbid condition is lingering in the epithelial lining of the alveoli. Whether or not this is dependent upon the constitutional infection which he presents is a question which has been discussed, and which we have endeavored to meet by adding iodide of potassium to the treatment, which, for the past few days, has consisted in the administration of carbonate of ammonia. The treatment of the acute stage consisted in the use of carbonate of ammonia, a moderate amount of quinine and stimulus in moderation.

The physical signs would be of interest, if I could demonstrate them to you. There has been, throughout the course of the case, low down on the right side, an area of unusual clearness on percussion, almost tympanitic. There was pseudo-tympany like that which is constantly found over a portion of the lung when the remainder is compressed by a pleural effusion, and which we sometimes find over the upper part of the lung when the lower part has become solid. The alteration of the tension of the vesicles, and in the pressure of the inspired air, give rise to a modification of resonance closely simulating that found over a cavity. To this modification, the name pseudo-tympany has been given to distinguish it from true tympany.

I indicated to you, in this case, the unusual distribution of the pneumonia. It began at the apex, and extended through to the back and downwards, until, perhaps, three-fourths of the right lung was involved, the lower part of the lower lobe in front remaining unaffected. It began as an apex pneumonia the posterior part next became affected, while the anterior part of the lower lobe remained intact. By far the majority of cases of pneumonia, present affection of the lower lobe, and in the majority of cases it remains limited to the lower lobe, but in a large number of cases, the disease extends from the lower to the upper lobe, and the whole lung becomes affected.

There are peculiarities about apex pneumonia to which I shall refer. It is far more eommon in children than in adults, and this occasionally leads to pneumonia in children being overlooked, from the failure to study the whole lung and the restriction of our attention more particularly to those points in which we are more apt to find consolidation in the adult. Not rarely little children will have true croupous pneumonia, running through its stages and terminating just as we see it in the adult, but limited throughout to the upper portion of one lung. Let me, in this connection, impress upon you the fact that there appear to be closer cerebral sympathies with this type of pneumonia than with the common basic pneumonia, and that partly because the nervous system of the child is extremely susceptible and partly from the reason that I have mentioned, there is apt to be developed cerebral symptoms of a marked type, so that this is known as the cerebral form of pneumonia, and these nervous symptoms are apt to still further obscure the recognition of the inflammation of the lung, and these cases are apt to be treated as cases of tubercular meningitis, or simple meningitis, and the pulmonary condition not recognized. In children with nervous symptoms, if cough or chest pain is noticed the chest should be examined with extreme care, front and back, from top to bottom. In these cases cerebral symptoms of the most alarming character may be present and pass away as the pneumonia diminishes.

Apex pneumonia is more common in young adults than it is either in children or mature people. It is apt to occur in those diposed to phthisis. There is trouble in securing complete resolution in such cases, which are apt to run into a sub-acute form and eventually develop into phthisis.

Again, apex pneumonia is met with under the influence of constitutional disturbances ; thus, when pneumonia appears as a complication of malarial fever, I have often seen it involve the apex. In typhoid fever, I have seen the inflammation involve the apex more frequently than is the case in frank, idiopathic pneumonia.

These are the three most important peculiarities of apex pneumonia: In the first place, its occurrence in a somewhat obscure form in children, being associated with marked cerebral symptoms. In the second place, its disposition to be followed by phthisis, and in the third place, its existence as a complication of some general specific disease.

I cannot say that syphilitic pneumonia, by which term I mean something different from pneumonia in the syphilitic, for those who have constitutional syphilis may have a frank pneumonia in the same way as one free from that taint, while, on the other hand, there is a special form of pneumonia which may be called syphilitic pneumonia, which is a syphilitic affection of the lungs with the infiltration of the tissue of the lungs with a special plasma, rich in epithelial cells, preventing, by its large amount and by the pressure which it exerts on the alveoli, the proper circulation of the blood, and giving rise to hepatization, which is very pale, dry and friable, being made up largely of epithelial elements, I cannot say that this syphilitic pneumonia especially involves the apex. It is as likely to affect the lower as the upper lobes.

I have already stated that there is, in the present case, an area over which pseudo-tympany is heard on percussion. In addition to this, careful percussion will develop at about the third interspace, a cracked pot sound. This is not to be attributed to a cavity, for none of the lung tissue has broken down· It is dependent on the fact that there still remains, at a considerable depth, infiltration and partial consolidation in the neighborhood of a large bronchial tube. This condition is similar to that which is present when there is a small cavity. By placing the body against a firm support, and percussing with more emphasis than usual, we communicate a shock to the air in the cavity, and express a part of it from the bronchus, giving rise to the peculiar chinking, which is known as the cracked pot sound. The same thing may be produced in certain conditions of partial consolidation in the neighborhood of a large bronchial tube, particularly if the ribs are at all flexible. The presence of this sound is one of the things that disturbs me in reference to this case, for it shows that while the morbid process has ended, there is still a deep infiltration of the lung, which is probably associated with the constitutional taint. I have no doubt that by a continuance of the treatment which I have mentioned, particularly by the use of specific remedies, we shall secure the removal of this infiltration, but it is a warning to us that although the temperature and pulse are normal, we should be careful how we allow these patients to expose themselves, until we are satisfied that the local conditions have entirely passed away.

We have all been taught, by sad experience, to be careful during the convalescence of certain specific diseases, notably typhoid fever, but I fear that we are not nearly so careful in the management of convalescence from local affections, particularly those of the chest. It is one thing for the temperature to fall to normal, the pulse to come down and the breathing become easy, and quite another thing for the local lesions to be entirely removed. Under such circumstances the patient, if allowed to expose himself, is in danger of a relapse. Even if a relapse does not take place, something which is worse may develop. If a slight trace of inflammatory process be overlooked and the patient be allowed to return to his ordinary occupation, it will remain and slowly take on a chronic degenerative change. The great majority of chronic troubles result from imperfectly cured local affections. This is pre-eminently true in regard to catarrhal pneumonia. It is true to a less degree as regards croupous pneumonia, and it is also true in regard to pleurisy. The criterion by which we are to judge when it is proper for the patient to rise, take exercise and expose himself, is solely the result of physical examination, showing that all trace of local disease has passed away. We cannot be governed by the general symptoms, for these may subside in a most satisfactory manner, and yet the patient be far from being entirely cured. The care which has been insisted on in the acute stage should never be relaxed until the physical examination shows that all local change has passed away, unless, after pursuing a judicious course, and keeping up this care for a reasonable time, we find that the patient, in consequence of some constitutional defect or peculiarity, is passing into a chronic stage. Under such circumstances further confinement, instead of being a benefit, would probably injure the constitution. The patient is then to be treated as one with a serious chronic disease, and although he is allowed to go about, it is under a most rigid hygienic regimen.

The consideration of the treatment of pneumonia demands more time than we can devote to it to-day. This man was treated in a way in which I think that you will treat most cases of this disease. When he was admitted, the disease had passed beyond the stage where depletion would be admissible. When the case is seen early, it is often well to use quite positive depletion, even if it is only local. In this case, there was no need for cardiac sedatives, but in many instances, when the patient is seen early, you will secure admirable results in limiting the inflammation and curtailing the inflammatory process by the use of veratrum viride or aconite. In order to assist the liquefaction of the exudation and stimulate expectoration, I know of no remedy equal to the carbonate of ammonia, especially if there is considerable vital depression. I consider quinine an almost essential element of the treatment of pneumonia, not in immense doses except when there is hyperpyrexia, but in doses of from eight to sixteen grains per day, given by the mouth if the stomach is perfectly tolerant, or by the rectum if it is not so. The diet is to be nutritious and the food given in small quantities and at short intervals. We are to be governed in the use of stimulants by the same considerations which control their use in other diseases. Many cases of pneumonia do very well without stimulants, and they should not be used as a matter of routine. We should wait for the development of symptoms, and when they are used, their effect should be carefully watched to see if they are doing what we wish before we continue them or increase the dose.

The treatment of this case will be continued as I have indicated, and I shall report the results.

